# The Coefficient of the Voltage Induced Frequency Shift Measurement on a Quartz Tuning Fork

**DOI:** 10.3390/s141121941

**Published:** 2014-11-19

**Authors:** Yubin Hou, Qingyou Lu

**Affiliations:** 1 High Magnetic Field Laboratory, Chinese Academy of Sciences, Hefei 230031, China; E-Mail: ybhou@hmfl.ac.cn; 2 Hefei National Laboratory for Physical Sciences at Microscale, University of Science and Technology of China, Hefei 230026, China

**Keywords:** qplus, quartz tuning fork, piezoelectric frequency effect, eigen-frequency shift, voltage induced frequency shift

## Abstract

We have measured the coefficient of the voltage induced frequency shift (VIFS) of a 32.768 KHz quartz tuning fork. Three vibration modes were studied: one prong oscillating, two prongs oscillating in the same direction, and two prongs oscillating in opposite directions. They all showed a parabolic dependence of the eigen-frequency shift on the bias voltage applied across the fork, due to the voltage-induced internal stress, which varies as the fork oscillates. The average coefficient of the VIFS effect is as low as several hundred nano-Hz per millivolt, implying that fast-response voltage-controlled oscillators and phase-locked loops with nano-Hz resolution can be built.

## 1. Introduction

The piezoelectric (PE) effect (including the reverse PE effect) refers to the approximately linear response of the mechanical and electrical mutual interactions in crystalline materials that lack inversion symmetry [1]. The importance of the PE effect is well known. One famous application utilizes the dimension change of the PE material under applied voltage to produce voltage controlled motion or positioning in real space. We can therefore call this type of PE effect the space domain PE effect. Its application examples are nano-positioners [2,3], piezoelectric motors [4–6], precise focusing of modern optical assemblies [7,8] and atomically and sub-atomically resolved scanning probe microscopes [9–16], *etc.* An obvious reason for these important applications is that the coefficient of the PE deformation is very low, around 0.1 micrometers per volt (or 1 Å per millivolt), providing very precise yet fast enough control of displacement.

Another particularly important application of the PE effect is the precise measurement of time, which exploits the ultrahigh stability of the eigen-frequency of a PE oscillator. We call this type of PE effect the frequency domain PE effect. Examples include quartz tuning forks in watches, crystal oscillators in computers, quartz crystal microbalances [17–20], voltage controlled oscillators (VCO), phase lock loops (PLL) [21], and cantilever excitors of atomic force microscopes [22–26]. However, the precise control or adjustment of the eigen-frequency of a PE oscillator is always an issue. The VCO's frequency shift is typically adjusted by an external capacitor whose capacitance is changed by the applied voltage on it. In this manner, the frequency stability is reduced and a high adjustment precision cannot be provided. For example, a 3.3 V voltage control crystal oscillator (VCXO) has a total pull range of 250 ppm and its initial frequency accuracy is typically within ±10 ppm [27], as the center frequency of the VCXO is usually several million hertz or even higher, it is not precise enough to be used for frequency measurements. Adding mass to the oscillator also can adjust the frequency shift, but this is not a controllable method. Zhou *et al.* have reported that the stress applied on a constrained quartz plate by a piezo-electric/piezo-magnetic ceramic plate can cause a very small frequency shift [28]. The frequency shift is of the order of 10^−5^ when the applied electric field is of the order of 100 V/mm or the magnetic field is of the order of 10 A/mm. This effect is small enough to be used in precise frequency control and measurement. However, the device is complicated since it uses an external piezo-electric/piezo-magnetic ceramic plate to control the stress.

Thus, a small voltage-induced frequency shift effect of the PE materials with simpler structures is urgently demanded in precise frequency control and measurement areas. This frequency domain PE effect could be as important as the space domain PE effect mentioned above, but nevertheless it has not been reported before.

Recently, quartz tuning forks (QTF) have been widely used as force sensors in scanning probe microscopes such as the atomic force microscope (AFM) and magnetic force microscope [29–31], in which the frequency shift of the QTFs is detected to measure the interaction between tip and sample.

In this paper, we will show that the frequency shift of a QTF can be controlled by a bias voltage applied on it, and the coefficient of this voltage induced frequency shift (VIFS) will be reported. The coefficient was found to be smaller than 1 mHz per volt (or sub micro-Hertz per millivolt). This extremely low value implies its immediate important applications in the high precision versions of VCO and PLL. Other possible applications include: a precise frequency sweeper, adjustable timing devices, and microbalances.

## 2. Experimental Setup

**Figure 1.**
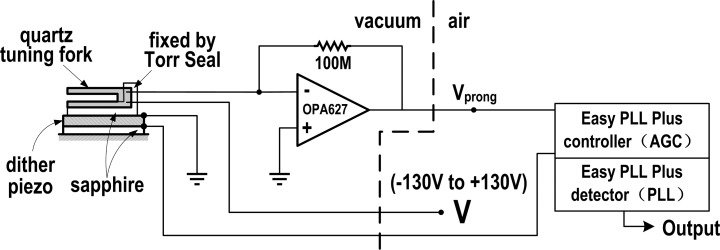
The schematic diagram of the setup employed to measure the VIFS effect.

Figure 1 shows the schematic diagram of the setup employed to measure the coefficient of the VIFS. One prong of the QTF (type E158 from Nanosurf AG, Liestal, Switzerland) was glued with Torr Seal on an L-shaped sapphire piece, which was in turn mounted by the same glue on a dither piezo plate. This type of QTF with one prong being fixed is often called qPlus QTF [31–33]. Another sapphire piece was sandwiched between the dither piezo and the base of the apparatus for good isolation. The oscillation details of the free prong of the QTF were measured by a current to voltage converter consisting of a 100 MOhm feedback resistor wired across an OPA627 operational amplifier (Texas Instruments, Dallas, TX, USA) with its input connected to the corresponding lead of the QTF [34]. When the free prong oscillates, the charge on it (due to the space domain PE effect) will change. The resultant current was converted into a voltage signal, Vprong, by the preamp and then connected back to the driving dither piezo via an automatic gain control, AGC, inside a commercial Easy PLL Plus controller by Nanosurf to form a closed loop oscillation circuit. The purpose of the AGC was to maintain constant oscillation amplitude.

A bias voltage (−130 to +130 V manually adjustable, generated from an SPM100 scanning probe microscope controller, RHK, Troy, MI, USA) was applied to the QTF between the virtual ground of the preamp and the remaining lead of the QTF. The oscillation loop ensured that the oscillation frequency tracked the QTF's instant eigen-frequency, so that we could measure the QTF's dynamic eigen-frequency and how it changed by measuring the oscillation frequency of the circuit loop with a PLL (Nanosurf Easy PLL Plus).

In the setup described above, only one prong of the QTF can oscillate. We can also vibrate both prongs in the same direction by using the dither piezo to vibrate the portion of the QTF where the two prongs join (*i.e.*, by mounting the joint on the dither piezo, not any of the prongs). This type of the mounted QTF is hereafter called in-phase QTF for simplicity. There is also the third vibration mode in which both prongs vibrate in the opposite direction (hereafter called anti-phase QTF). To this end, the setup is similar to that of the in-phase QTF except that the dither piezo is removed and the oscillation driving signal is connected to the non-inverting input of the preamp (the joint of the prongs is fixed on the base). In the work reported here, the qPlus, in-phase and anti-phase vibration modes were all studied and compared.

## 3. Results and Theoretical Explanation

**Figure 2.**
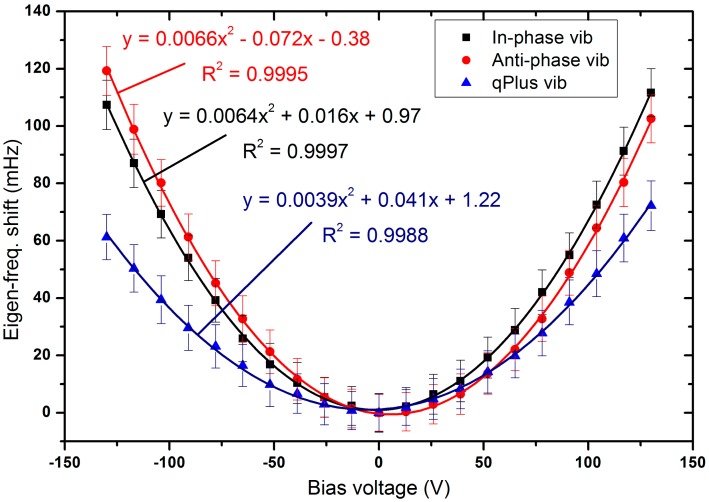
Plots of the eigen-frequency shifts *vs.* the applied bias voltages for the three different prong vibration modes. Error bars: ±1 standard deviation. The data of each vibration mode can be fitted well with a parabolic curve.

The measured dependences of the eigen-frequency shifts of the qPlus, in-phase and anti-phase QTF on the bias voltage applied to the QTF are shown in Figure 2. Each is excellently fit by a quadratic function. The fitted parabolic curves approximately pass through the origin, so they can be written in the form *y* = a*x*^2^ with a = 0.0039, 0.0066, and 0.0064 mHz/V^2^, respectively. Since the eigen-frequency shift is not linearly dependent on the bias voltage, the VIFS coefficient is not a constant. Nevertheless, we can still discuss its average VIFS coefficient defined as the eigen-frequency change divided by the bias voltage change.

The average VIFS coefficients were extremely low and were 470, 917 and 826 nano-Hz per millivolt for the qPlus, in-phase and anti-phase QTFs, respectively, implying that the most precise nano-Hz resolution VCOs and PLLs can be built based on the VIFS effect.

**Figure 3.**
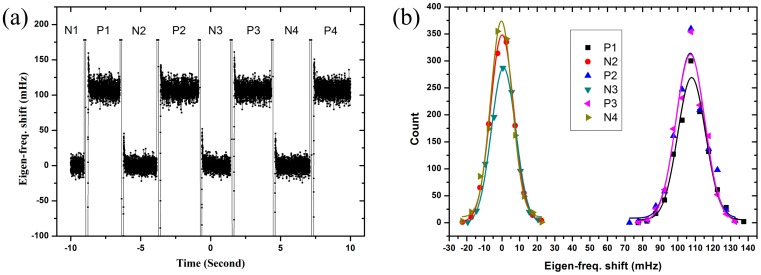
(**a**) The plot of the eigen-frequency change as the bias voltage was switched between 0 V and +130 V for the anti-phase QTF. The results of the in-phase and qPlus QTFs were very similar and they are omitted here; (**b**) Gaussian distributions fitted to the data. The bias voltages at 0 V and +130 V are labelled N and P, respectively.

To check the repeatability of the VIFS of the three QTF modes, we mechanically switched the bias voltages on the three types of QTF repeatedly between 0 V and +130 V and watched the output signals of the Easy PLL Plus. It turned out that they all looked similar. As an example, the step response of the anti-phase QTF is shown in Figure 3a. The big fluctuations between high and low outputs are apparently due to the mechanical switching. Figure 3b is the Gaussian distributions of the data (the data of the big fluctuations due to the mechanical switching were removed). It shows that the data with bias voltages of 0 V and +130 V separated completely. The center values of the frequency shifts at low and high bias voltages are both very close, implying the excellent repeatability. All the experiments were done in low vacuum and room temperature.

**Figure 4.**
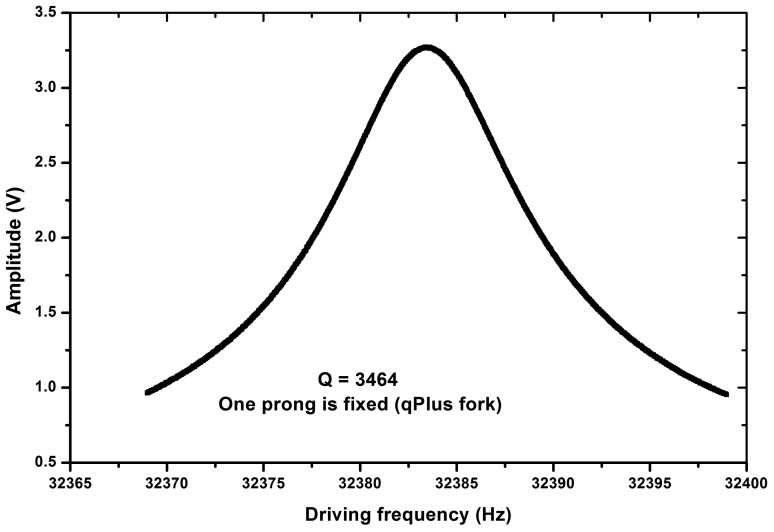
The resonance curve of the qPlus QTF used in the experiment. It is fairly symmetric with a Q factor equal to 3464.

The resonance curve for the qPlus QTF is shown in Figure 4. It is rather symmetric. The curves are a little skewed for the in-phase and anti-phase QTFs. The Q values are all above 3000, which is typical for QTFs in low vacuum and room temperature, showing that the QTFs were functioning well.

To explain why the VIFS effect can happen, we notice that applying a bias voltage *V* on the QTF will produce an electrostatic force on the QTF just as applying a voltage on a capacitor will exert an electrostatic force on the dielectric. This force *F_V_* is given by:

$$F_{V}=CV^{2}/2d$$

where *C* is the capacitance of the QTF in its model and *d* is the effective separation between the electrodes of the capacitor. Before the bias voltage is applied, the free oscillation (presumably in the *x* direction) of the QTF experiences only a restoring force of *F*_0_ = –*k*_0_*x*. After the bias voltage is turned on, *d* is a function of the applied voltage *V* and *x* and the force on the QTF becomes:

$$\begin{array}{ll} F_{1} & =-k_{0}x+F_{V}\\ & =-k_{0}x+CV^{2}/2d\left(V,x\right)\\ & =-k_{0}x+\beta V^{2}/{\left[d\left(V,x\right)\right]}^{2} \end{array}$$

where *C* is assumed to be proportional to [*d*(*V*,*x*)]^–1^ and *β* contains all the constants in the last term.

The Young's modulus of quartz is 7.87×10^11^*N*/*m*^2^ [29], which is large, meaning that the change of *d* under *V* is small. The change of *d* due to *x* is also small. Therefore, *d*(*V*,*x*) can be expanded in a Taylor series, and *F*_1_ can be re-written as:

$$\begin{array}{ll} F_{1} & =-k_{0}x+\beta V^{2}\cdot {\left[d\left(V,x\right)\right]}^{-2}\\ & =-k_{0}x+\beta V^{2}\cdot \left[{\left[d\left(V_{0},x_{0}\right)\right]}^{-2}+{\frac{\partial {\left[d\left(V,x\right)\right]}^{-2}}{\partial d\left(V,x\right)}\cdot \frac{\partial d\left(V,x\right)}{\partial V}|}_{\begin{array}{c} V=V_{0}\\ x=x_{0} \end{array}}\cdot \left(V-V_{0}\right)+{\frac{\partial {\left[d\left(V,x\right)\right]}^{-2}}{\partial d\left(V,x\right)}\cdot \frac{\partial d\left(V,x\right)}{\partial x}|}_{\begin{array}{c} V=V_{0}\\ x=x_{0} \end{array}}\cdot \left(x-x_{0}\right)+...\right]\\ & =-k_{0}x+\beta V^{2}\cdot \left[{\left[d\left(V_{0},x_{0}\right)\right]}^{-2}-{\frac{2}{{\left[d\left(V,x\right)\right]}^{3}}\cdot \frac{\partial d\left(V,x\right)}{\partial V}|}_{\begin{array}{c} V=V_{0}\\ x=x_{0} \end{array}}\cdot \left(V-V_{0}\right)-{\frac{2}{{\left[d\left(V,x\right)\right]}^{3}}\cdot \frac{\partial d\left(V,x\right)}{\partial x}|}_{\begin{array}{c} V=V_{0}\\ x=x_{0} \end{array}}\cdot \left(x-x_{0}\right)+...\right]\\ & =-\left(k_{0}+C_{3}\beta V^{2}\right)\cdot x+\beta V^{2}\cdot \left[C_{1}-C_{2}\cdot \left(V-V_{0}\right)+C_{3}\cdot x_{0}+...\right] \end{array}$$

where *C*_1_ = [*d*(*V*_0_,*x*_0_)]^–2^, ${C_{2}=\frac{2}{{\left[d\left(V,x\right)\right]}^{3}}\cdot \frac{\partial {\left[d\left(V,x\right)\right]}^{2}}{\partial V}|}_{\begin{array}{c} V=V_{0}\\ x=x_{0} \end{array}}$ and ${C_{3}=\frac{2}{{\left[d\left(V,x\right)\right]}^{3}}\cdot \frac{\partial {\left[d\left(V,x\right)\right]}^{2}}{\partial x}|}_{\begin{array}{c} V=V_{0}\\ x=x_{0} \end{array}}$ are constants.

Note that *V* is a constant which is determined by the voltage source. Any terms that do not vary with *x* should not cause the force constant (and of course the eigen-frequency) of the QTF to change. Thus, the dynamic force constant becomes *k*_0_+*C*_3_*βV*^2^. The dynamic resonant frequency becomes:

$$\begin{array}{ll} \omega & =\sqrt{\frac{k_{0}+C_{3}\beta V^{2}}{m}}\\ & =\sqrt{\frac{k_{0}}{m}}\cdot {\left(1+\frac{C_{3}\beta V^{2}}{k_{0}}\right)}^{\frac{1}{2}} \end{array}$$

where *m* is the effective mass of the QTF. Since the frequency shift is very small, *ω* can be approximately written as:

$$\omega \approx \sqrt{\frac{k_{0}}{m}}\cdot \left(1+\frac{C_{3}\beta V^{2}}{2k_{0}}\right)$$

which is a parabolic function of *V*, thus consistent with our measured results. From this interpretation, we also know that response of the frequency domain PE effect should be as fast as the space domain PE effect because they stem from the same cause.

## 4. Conclusions

In summary, we have measured the VIFS effect on three vibration modes of the QTFs. Owing to the voltage-induced internal stress, which varies as the fork oscillates, the eigen-frequency shifts in term of a parabolic function of the voltage applied on the QTFs. Since the time domain VIFS effect and the space domain PE effect have the same cause, they will have the same response time. The average coefficient of the VIFS is very small (several hundred nano-Hz per millivolt). From this, we expect that the most precise (nano-Hz) yet fast-responding voltage-controlled oscillators and phase-locked loops can be built.
